# RETRACTED: Di Paola et al. Environmental Risk Assessment of Dexamethasone Sodium Phosphate and Tocilizumab Mixture in Zebrafish Early Life Stage (*Danio rerio*). *Toxics* 2022, *10*, 279

**DOI:** 10.3390/toxics13020085

**Published:** 2025-01-24

**Authors:** Davide Di Paola, Jessica Maria Abbate, Carmelo Iaria, Marika Cordaro, Rosalia Crupi, Rosalba Siracusa, Ramona D’Amico, Roberta Fusco, Daniela Impellizzeri, Salvatore Cuzzocrea, Nunziacarla Spanò, Enrico Gugliandolo, Alessio Filippo Peritore

**Affiliations:** 1 Department of Chemical, Biological, Pharmaceutical, and Environmental Science, University of Messina, 98166 Messina, Italy; dipaolad@unime.it (D.D.P.); ciaria@unime.it (C.I.); rsiracusa@unime.it (R.S.); rdamico@unime.it (R.D.); rfusco@unime.it (R.F.); dimpellizzeri@unime.it (D.I.); aperitore@unime.it (A.F.P.); 2 Department of Veterinary Science, University of Messina, 98166 Messina, Italy; jessica.abbate@unime.it (J.M.A.); rcrupi@unime.it (R.C.); egugliandolo@unime.it (E.G.); 3 Department of Biomedical and Dental Sciences and Morphofunctional Imaging, University of Messina, 98166 Messina, Italy; cordarom@unime.it; 4 Department of Pharmacological and Physiological Science, Saint Louis University School of Medicine, Saint Louis, MO 63104, USA

The journal retracts the article “Environmental risk assessment of mixture of COVID-19 treating pharmaceutical drugs in zebrafish early life stage (*Danio rerio*)” [1], cited above.

Following publication, concerns were brought to the attention of the Editorial Office regarding the duplication of images contained within this publication [1]. 

Adhering to our complaints procedure, an investigation was conducted by the Editorial Office and the Editorial Board that confirmed the duplication of a number of panels contained within Figures 1 and 3 presenting different experimental conditions. Following discussions with the authors, the Editorial Board were unable accept the explanation provided or verify the validity of the above-mentioned images. Consequently, the Editorial Board has lost confidence in the reliability of the findings and decided to retract this publication [1], as per MDPI’s retraction policy (https://www.mdpi.com/ethics#_bookmark30).

This retraction was approved by the Editor-in Chief of the journal *Toxics*. 

The authors did not agree to this retraction.
